# Microfluidic-Assisted ZIF-Silk-Polydopamine Nanoparticles as Promising Drug Carriers for Breast Cancer Therapy

**DOI:** 10.3390/pharmaceutics15071811

**Published:** 2023-06-24

**Authors:** Zijian Gao, Muhamad Hawari Mansor, Natalie Winder, Secil Demiral, Jordan Maclnnes, Xiubo Zhao, Munitta Muthana

**Affiliations:** 1 Department of Oncology and Metabolism, University of Sheffield, Beech Hill Road, Sheffield S10 2RX, UK; 2 Department of Chemical and Biological Engineering, University of Sheffield, Beech Hill Road, Sheffield S10 2RX, UK; 3 School of Pharmacy, Changzhou University, Changzhou 213164, China

**Keywords:** microfluidic-assisted, ZIF-8, silk fibroin, polydopamine, curcumin, nanoparticles, controlled release, MDA-MB-231, SK-BR-3, MCF-7, AD-293

## Abstract

Metal–organic frameworks (MOFs) are heralded as potential nanoplatforms for biomedical applications. Zeolitic imidazolate framework-8 (ZIF-8), as one of the most well known MOFs, has been widely applied as a drug delivery carrier for cancer therapy. However, the application of ZIF-8 nanoparticles as a therapeutic agent has been hindered by the challenge of how to control the release behaviour of anti-cancer zinc ions to cancer cells. In this paper, we designed microfluidic-assisted core-shell ZIF-8 nanoparticles modified with silk fibroin (SF) and polydopamine (PDA) for sustained release of zinc ions and curcumin (CUR) and tested these in vitro in various human breast cancer cells. We report that microfluidic rapid mixing is an efficient method to precisely control the proportion of ZIF-8, SF, PDA, and CUR in the nanoparticles by simply adjusting total flow rates (from 1 to 50 mL/min) and flow rate ratios. Owing to sufficient and rapid mixing during microfluidic-assisted nanoprecipitation, our designer CUR@ZIF-SF-PDA nanoparticles had a desired particle size of 170 nm with a narrow size distribution (PDI: 0.08), which is much smaller than nanoparticles produced using traditional magnetic stirrer mixing method (over 1000 nm). Moreover, a properly coated SF layer successfully enhanced the capability of ZIF-8 as a reservoir of zinc ions. Meanwhile, the self-etching reaction between ZIF-8 and PDA naturally induced a pH-responsive release of zinc ions and CUR to a therapeutic level in the MDA-MB-231, SK-BR-3, and MCF-7 breast cancer cell lines, resulting in a high cellular uptake efficiency, cytotoxicity, and cell cycle arrest. More importantly, the high biocompatibility of designed CUR@ZIF-SF-PDA nanoparticles remained low in cytotoxicity on AD-293 non-cancer cells. We demonstrate the potential of prepared CUR@ZIF-SF-PDA nanoparticles as promising carriers for the controlled release of CUR and zinc ions in breast cancer therapy.

## 1. Introduction

Nanotechnology, as one of the most potent techniques for cancer therapy, has attracted significant attention in recent decades. Various nanomaterials, such as liposomes, polymers, micelles, dendrimers, proteins, and inorganic nanoparticles, have been developed in significant cancer treatment fields, such as chemotherapy, gene therapy, radiotherapy, and immunotherapy [1,2,3]. Among various types of cancer, breast cancer remains one of the most common causes of cancer-related death among female patients [4,5]. Nanomedicine encapsulated with diverse anticancer drugs, including doxorubicin, cisplatin, and fluorouracil, and modified with therapeutic peptide/protein, have been used as multifunctional theranostic systems in breast cancer treatment [6,7,8]. Currently, some nanoplatforms, such as PEGylated liposomal doxorubicin hydrochloride and albumin-bound paclitaxel nanoparticles as breast cancer drugs, have been approved by the FDA, indicating a large potential of nanomedicine in breast cancer therapy [9].

To date, various methods have been used to synthesize nanoparticles with a desired size, shape, structure, and surface modification to meet numerous therapeutic requirements [10,11,12,13]. However, traditional nanoparticle preparation methods, such as breaking down large particles and self-assembly of monomers, suffer from wide size distribution, tedious synthesis processes, and large batch-to-batch variability [14,15]. Compared with other methods, although traditional nanoprecipitation dramatically simplifies the process of nanoparticles preparation and reduces the size distribution, due to a lack of accurate control on mixing time and flow regime during the nanoprecipitation process, there is still abundant room for optimizing mixing parameters to obtain well controlled particle properties [16,17] Recently, the microfluidic approach has emerged as a technology, manipulating tiny fluids in microchannels with the dimension of tens of micrometres to synthesize stable and controllable nanomaterials in a simple procedure [18]. Several types of microfluidic devices, including T or Y-junction mixers, co-flowing junction mixers, hydrodynamic flow focusing mixers, vortex mixers, and staggered herringbone mixers, have been applied in nanoparticle production by accurate handling of flow patterns [17]. Compared with traditional nanoprecipitation methods, a microfluidic-assisted nanoprecipitation method can provide rapid and adequate mixing during nanoprecipitation in which one solution containing particle materials meets another anti-solvent in a certain short time (around several milliseconds) so that the triggering of nucleation and growth of nanoparticles will be under precise control. In addition, easily maintaining uniform reaction conditions and fewer regents’ consumption ensure its accuracy, reproducibility, and low running cost on the fabrication of nanoparticles [19,20,21,22].

In our previous studies, we designed a novel microfluidic swirl mixer, which was used to successfully optimize the synthesis of silk and lipid nanoparticles [23,24,25], indicating its potential for developing new nanoformulations as an effective multifunctional drug delivery system. Metal–organic frameworks (MOFs), consisting of metal ions and organic ligands, have been considered as potential drug carriers because of their diverse structures, large surface-to-volume ratios, controllable pore size, and great biocompatibility [26,27,28,29]. Zeolitic imidazolate framework-8 (ZIF-8), made up of a zinc ion centre and 2-methylimidazolate linkers, is one of the most widely used groups of MOFs in drug delivery systems, owing to its high loading efficiency and easy modification [30]. Appropriate pH responsiveness gives ZIF-8 nanoparticles a pH-controlled drug release property [31]. In addition, recent evidence suggests that zinc ions released from ZIF-8 nanoparticles could promote the generation of reactive oxygen species (ROS), which could be used to induce apoptosis, autophagy, necroptosis, DNA damage, and reduce multidrug resistance of cancer cells [32,33,34]. To further control the release behaviour of zinc ions, polydopamine (PDA), a widely used bioinspired material, has been coated on the ZIF-8 nanoparticles as a self-etching induced shell [35]. The inherent zinc ions’ chelating ability allows PDA coating to efficiently etch the ZIF-8 core and speed up the release rate of zinc ions to meet anticancer requirements [36]. 

However, previously prepared PDA-coated ZIF-8 nanoparticles were not very efficient zinc ions reservoirs. Solution oxidation, as the most simply used PDA coating method, requires an alkaline environment (pH > 7.5), adequate oxidants, and a long polymerization time, where the ZIF-8 template suffers from hydrolysis-induced degradation and prolonged etching with PDA precursors and its derivatives, leading to the loss of large portions of zinc ions during the PDA coating process [35,37]. To minimise the unnecessary loss of zinc ions and to adjust the etching process, materials, such as silica, have been tried to build intermediate protection layers between the ZIF-8 core and PDA coating [38]. Nevertheless, there is still abundant room for developing an appropriate intermediate layer to improve the release pattern of zinc ions. 

Silk as a natural material has been generally used in the fabric industry. Recently, due to its biocompatibility and low immunogenicity, silk fibroin (SF)-related nanoparticles show great potential in developing alternative carriers for anticancer drugs [39]. SF arranges itself in three different forms, including silk I, silk II, and silk III. Among them, silk II contains an antiparallel β-sheet/crystal molecular structure, allowing high-temperature stability and both water and solvent insolubility [40,41,42]. Excellent biocompatibility and high stability greatly increase the value of SF as a potential intermediate protection layer to control the etching reaction between zinc ions and catechol groups from PDA. 

Curcumin (CUR) is a natural representative polyphenol extracted from the curcuma longa plant, with multiple anticancer activities [43]. In breast cancer treatment, CUR has proven ability to inhibit the proliferation of cancer cells by inhibiting key targets, such as NF-κB inducing genes, alterations in the protein kinase B (Akt), and human epidermal growth factor receptor 2 (HER2) [44]. Despite CUR’s significant anticancer activities, its use as a pharmaceutical agent has been limited by low aqueous solubility, rapid metabolism, and poor absorption. To address these limitations, numerous nano-drug delivery systems, including liposomes, polymeric nanoparticles, protein nanoparticles, solid–lipid nanoparticles, metal nanoparticles, and nano-emulsion, have been developed and demonstrated the ability to significantly improve the therapeutic efficiency of CUR by enhancing its bioavailability and targetability [45,46,47,48,49,50]. Table 1 provides some examples of nano-formulation of CUR in breast cancer treatment. In this work, for the first time, we designed and produced a core-shell microfluidic-assisted ZIF-8 nanoparticle protected by SF as an intermediate layer and coated by PDA for zinc ion release. A special four-element swirl microfluidic chip was used to provide rapid and sufficient mixing during ZIF-8 nanoprecipitation and free adjustment of the SF intermediate layer, resulting in a controllable zinc ion release framework. Compared with the traditional mixing method, the microfluidic-assisted method successfully reduced nanoparticles size from 1000 nm to 170 nm, which is more desirable for penetrating tumour blood vessels—enhanced permeability and retention (EPR) effect [51]. In addition, we encapsulated CUR as a hydrophobic anti-cancer drug into our ZIF-based nanoparticles (CUR@ZIF-SF-PDA) to achieve a pH-responsive drug and zinc ion delivery system. CUR@ZIF-SF-PDA nanoparticles demonstrated enhanced cellular uptake efficiency and anticancer properties in various breast cancer cells and retained low cytotoxicity in relation to non-cancer cells at the same time.

## 2. Materials and Methods

### 2.1. Materials

Bombyx mori silk was purchased from Jiangsu, China. Na_2_CO_3_ (11552), DMSO (dimethyl sulfoxide, A13280), curcumin (B21573.09), and dopamine hydrochloride (A11136) were purchased from Alfa Aesar, Haverhill, MA, USA. Methanol (34860), CaCl_2_ (C1016), C_2_H_5_OH (ethanol, 51975), 2-Methylimidazole (M50850), a zinc assay kit (MAK032-1KT), a dialysis tubing cellulose membrane (D9777-100FT), paraformaldehyde (158127), and hydrochloric acid (7647-01-0) were purchased from Sigma-Aldrich, St. Louis, MO, USA. PI/RNAse Staining Solution, Zinc nitrate hexahydrate (228737), Tris Base (BP152-1), MTT (M6494), DAPI (D1306), DiD (V22887), and foetal bovine serum (FBS) were purchased from Fisher Scientific, Waltham, MA, USA. PBS (BE17-512F), RPMI-1640, DMEM (−)Pyruvate and DMEM (+)Pyruvate were purchased from Lonza, Basel, Switzerland. Human Caucasian breast adenocarcinoma cells (MDA-MB-231) and AD-293 human embryonic kidney cells were purchased from ECACC, Salisbury, UK. Human breast cancer cells (SK-BR-3 and MCF-7) were purchased from ATCC, Manassas, VA, USA.

### 2.2. Preparation of 4-Elements Swirl Microfluidic Device

**Figure 1:**
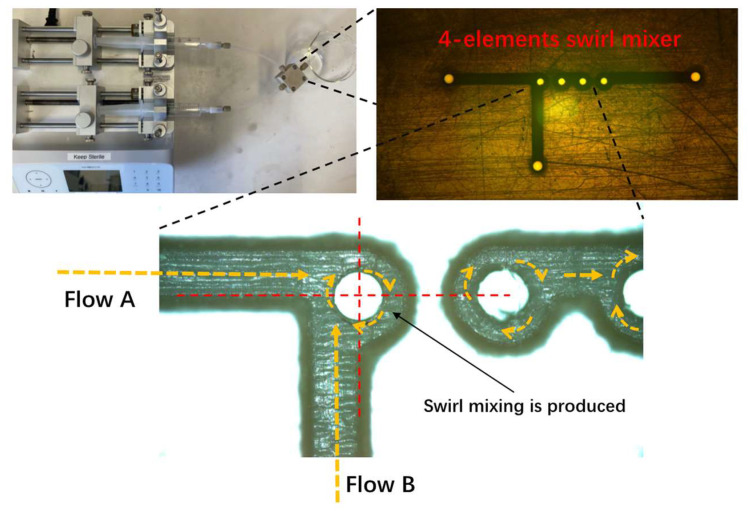
Schematic diagram of the preparation for microfluidic-assisted rapid mixing.

Figure 1 shows the set-up of the microfluidic device with a 4-element swirl mixer. The volume of a single mixing element is 0.000612 mL. Solution A and solution B were filled in two separate 20 mL syringes powered by a dual syringe pump (Fusion 4000, Chemyx Inc., Stafford, TX, USA). Two syringe inlet channels were connected to a series of the swirl mixing elements, in which the flow centre of inlet channels deviated from the centre of the mixing element, therefore rapid swirl mixing was generated. Two blocks with O-rings were used to seal the surface of the mixers, thus a completed mixing system was achieved. Reynolds number (Re) was calculated to identify different flow regimes (laminar, transitional, or turbulent flow) by Equation (1), where the fluid density (ρ) and viscosity (μ) are averages of the values in the two unmixed feed solutions, u is average velocity through the holes connecting the swirl chambers, and $D_{h}$ is the connecting hole diameter [57]. The flow mixing time ($\tau_{m}$) is the mean residence time of fluid passing through the mixer from the inlets to the downstream section and was calculated by Equation (2), where $v_{m}$ is the volume of a single mixing element, and $Q_{total}$ is the total flow rate of mixing fluid.

(1) $$Re=\frac{\rho uD_{h}}{\mu }$$

(2) $$\tau_{m}=\frac{v_{m}}{Q_{total}}$$

### 2.3. Preparation of SF Solution

SF solution was extracted from Bombyx mori silk following the previous method [58]. Briefly, 5 g silk was cut into small pieces and boiled in water (2 L) containing 0.02 M sodium carbonate for 30 min. The resulting silk was removed from the solution and rinsed with UHQ water at least 3 times until the solution was clear. After drying overnight, 2 g degummed silk was dissolved in 20 g filtered Ajisawa’s reagent (1:2:8 molar ratio of CaCl_2_: Ethanol: Deionised (DI) water) at 75 °C for 3 h. The collected SF solution was dialysed in a cellulose dialysis tube (12 KDa cut off) against UHQ water for 3 days to remove the remaining CaCl_2_ and ethanol. The final solution was centrifuged twice (13,000 rpm, 10 min) to remove the impurities, and it was stored at 4 °C.

### 2.4. Synthesis of MOF (ZIF-8) Nanoparticles

Microfluidic-assisted ZIF-8 nanoparticles were prepared by separately mixing 5, 10, and 15 mL 2-Methylimidazole (2-MI) solution (33 mg/mL, methanol as solvent) with 5 mL zinc nitrate hexahydrate (ZIN) solution (30 mg/mL, DI water as solvent) through the microfluidic device with various total flow rate (1, 5, 10, 25, and 50 mL/min). The as-prepared ZIF-8 nanoparticles were collected by centrifuging at 13,000 rpm for 15 min. Finally, the product was washed with DI water and stored at −20 °C. A sonicator with a probe (Vibra cell CV18, Sonics & Materials, Newtown, CT, USA) was used to resuspend nanoparticles before further analysis. To identify the effect of different solvents on the size change in ZIF-8 nanoparticles, various solvents, including DI water, ethanol, and methanol, were used during the synthesis.

### 2.5. Synthesis of ZIF-8 Based Core-Shell Drug Delivery Nanoparticles (CUR@ZIF-SF-PDA)

CUR-loaded ZIF-8 nanoparticles (CUR@ZIF) were simply prepared by dissolving 5 mg CUR in 10 mL 2-MI solution (33 mg/mL, methanol as solvent) and then mixed with 5 mL ZIN solution (30 mg/mL, DI water as solvent) through the microfluidic device. The collection process was the same as previously described in Section 2.4. After that, 3.7 mg prepared CUR@ZIF nanoparticles were dispersed in 1 mL DI water, and then 1 mL SF solution with different concentrations (0.75, 1.1, and 1.25 mg/mL, DI water as solvent) were mixed with above solution through the microfluidic device to prepare SF protected nanoparticles (CUR@ZIF-SF). Finally, to produce PDA-coated self-etching nanoparticles (CUR@ZIF-SF-PDA), 1.25 mg dopamine hydrochloride was added to 1 mL of the previously prepared SF solution before mixing with the CUR@ZIF suspension. After microfluidic mixing, the obtained mixture was kept at room temperature with gentle stirring for 1 h. After the reaction finished, CUR@ZIF-SF-PDA nanoparticles were isolated and purified, as previously described in Section 2.4. ZIF-PDA nanoparticles were prepared by the same method without CUR loading and SF coating. A sonicator with a probe was used to resuspend nanoparticles before further analysis. As a comparison, instead of using the microfluidic device, the normal preparation method relied on traditional mixing through a magnetic stirrer.

### 2.6. Particles Characterization

#### 2.6.1. Size and Zeta Potential Analysis

The size and Zeta potential of CUR@ZIF-SF-PDA were measured by Dynamic Light Scattering (DLS) (NanoBrook 90 plus Pals Particle size Analyser, Brookhaven Instrument, Upton, NY, USA). Nanoparticles were dispersed in filtered DI water in a cuvette. The wavelength of the diode laser was set at 660 nm, and the temperature was kept at 25 °C. Refractive indexes were set as 1.3 and 1.6 for water and nanoparticles, respectively. Three batches of samples were prepared and analysed. Polydispersity Index (PDI) was calculated as PDI = (σ/d)^2^, in which σ is the standard deviation, and d is the mean particle diameter [59]. The PDI was automatically calculated after each test through DLS. The stability of desired CUR@ZIF-SF-PDA nanoparticles was analysed during 5 days of storage at −20 °C.

#### 2.6.2. Morphological Analysis

The morphological properties of designed nanoparticles were characterised by transmission electron microscopy (TEM) and scanning electron microscopy (SEM). During TEM analysis, 10 µL of suspension of nanoparticles was placed on a carbon-coated grid for 1 min and dried with a filter paper before imaging. TEM images were obtained from the FEI Tecnai G2 Spirit BioTWIN with accelerating voltage at 80 kV. SEM samples were prepared by placing 2 mg of dried nanoparticles on SEM specimen stubs and coated with 10 nm Au. SEM images were obtained from FEI Inspect F50 Microscope operated at 10 kV.

#### 2.6.3. Fourier Transform Infrared Spectroscopy (FTIR) Analysis

The chemical composition and functional groups of designed nanoparticles were investigated by FTIR (IR Prestige-21, Shimadzu, Kyoto, Japan) with a scan range from 400 to 4000 cm^−1^. The spectrum data were analysed by Happ-Genzel apodization over 64 scans with a resolution of 4 cm^−1^.

### 2.7. Encapsulation and Loading Efficiency of CUR and Zinc Ions

The concentrations of zinc ions and CUR were identified by zinc assay kit and UV-Vis spectrometry (JENWAY 6715, Bibby Scientific, Staffordshire, UK). The standard calibration curves of CUR and zinc ions were obtained by measuring the maximum peak absorbance at 435 nm and 560 nm, respectively. Encapsulation efficiency and loading efficiency were calculated as below:

$$Encapsulation\;efficiency\left(w/w\%\right)=\frac{amount\;of\;CUR\;or\;zinc\;ions\;in\;particles}{amount\;of\;CUR\;or\;zinc\;ions\;initially\;added}\times 100\%$$

$$Loading\;efficiency\left(w/w\%\right)=\frac{amount\;of\;CUR\;or\;zinc\;ions\;in\;particles}{amount\;of\;total\;particles}\times 100\%$$

### 2.8. In Vitro pH-Responsive CUR Release Analysis

To study the CUR release profile, 1 mg designed nanoparticles were suspended in 1 mL PBS/Ethanol (50% *v*/*v*, pH= 7.4, 6.5, and 5.5) at 37 °C with shaking at 200 rpm. After that, the suspension was centrifuged (13,000 rpm, 20 min) at certain intervals, and the supernatant was removed and replenished with fresh release medium. The CUR concentration of each supernatant was calculated from the UV-Vis standard calibration curve.

### 2.9. In Vitro SF/PDA-Controlled Zinc Ion Release Analysis

To identify the influence of the SF intermediate layer and PDA coating on the release behaviour of zinc ions, 1 mg ZIF-8-based nanoparticles with different concentrations of SF were suspended in 1 mL PBS (pH = 7.4, 6.5, and 5.5) at 37 °C with shaking at 200 rpm. After that, the supernatant was separated as previously described, and then the release profile of zinc ions was quantified by a zinc assay kit (Sigma-Aldrich, St. Louis, MO, USA).

### 2.10. Cellular Uptake Analysis

MDA-MB-231 breast cancer cells and AD-293 human embryonic kidney cells were cultured in DMEM (−)Pyruvate and DMEM (+)Pyruvate medium supplemented with 10% Foetal Bovine Serum (FBS), 1% Penicillin/Streptomycin, 1% L-glutamine, and 5% CO_2_ at 37 °C. After that, MDA-MB-231, and AD-293 cells were seeded in 12-well plates at a density of 1 × 10^5^ cells per well and incubated overnight. Then, the cells were treated with 100 µg/mL of CUR@ZIF, CUR@ZIF-SF, CUR@ZIF-SF-PDA nanoparticles, and free CUR (Ex 488 nm, Em 550 nm) for 24 h. The concentrations of CUR were maintained at the same level during each treatment. To obtain confocal fluorescence microscopy images, treated cells were washed with PBS three times and fixed with 4% paraformaldehyde for 20 min. Next, the fixed cells were washed with PBS again and stained with DAPI (Ex 350 nm, Em 470 nm) and DiD (Ex 644 nm, Em 663 nm) for 20 min. Fluorescent images of treated cells were captured by a Zeiss LSM 980 microscope (Zeiss, Oberkochen, Germany).

### 2.11. Biocompatibility and In Vitro Cytotoxicity Analysis

MDA-MB-231, SK-BR-3, MCF-7, and AD-293 cells were cultured, as previously described, in DMEM (−)Pyruvate, RPMI-1640, DMEM (−)Pyruvate, and DMEM (+)Pyruvate media, respectively. The MTT assay was used to detect the cytotoxicity of ZIF-8, CUR@ZIF, CUR@ZIF-SF, and CUR@ZIF-SF-PDA nanoparticles. During the test, cells were seeded into 96-well plates at a density of 2500 cells per well (100 µL) and incubated overnight. After that, the medium was removed and replaced with a fresh medium, and different nanoparticles were put in each well to reach final concentrations of 0, 25, 50, 100, and 200 µg/mL. After 24, 48, and 72 h of incubation, 10 µL of 12 mM MTT was added to each well and incubated at 37 °C for 4 h, followed by removing the 85 µL supernatants and adding 50 µL DMSO. The absorbance of each well was measured at 540 nm. The cell viability was measured by comparing the absorbance with control wells.

### 2.12. Cell Cycle Analysis

SK-BR-3, MCF-7, and AD-293 cells were cultured at 2.5 × 10^5^ cells/mL in a 6-well plate, as previously described, and incubated overnight. Then, the cells were treated with 100 µg/mL of ZIF-8, CUR@ZIF, CUR@ZIF-SF, and CUR@ZIF-SF-PDA nanoparticles, as well as free CUR, for 24 h. After that, the treated cells were fixed in cold methanol at −20 °C for at least 2 h. Next, 1 × 10^6^ fixed cells were washed and mixed with 0.5 mL of PI/RNAse solution. Stained cells were incubated for 30 min at room temperature in the dark, and a flow cytometer (BD FACSCanto^TM^ II, Franklin Lakes, NJ, USA) was used to determine the cell cycle stage. 

### 2.13. Statistical Analysis

Statistical analysis was performed using One-way analysis of variance through GraphPad Prism 9 software (GraphPad Software Inc., La Jolla, CA, USA). In all comparisons, statistical significance was determined at *p* ≤ 0.05.

## 3. Results and Discussion

### 3.1. Microfluidic-Improved Synthesis of ZIF-8-Based Nanoparticles

#### 3.1.1. Microfluidic-Improved Rapid Mixing

**Figure 2:**
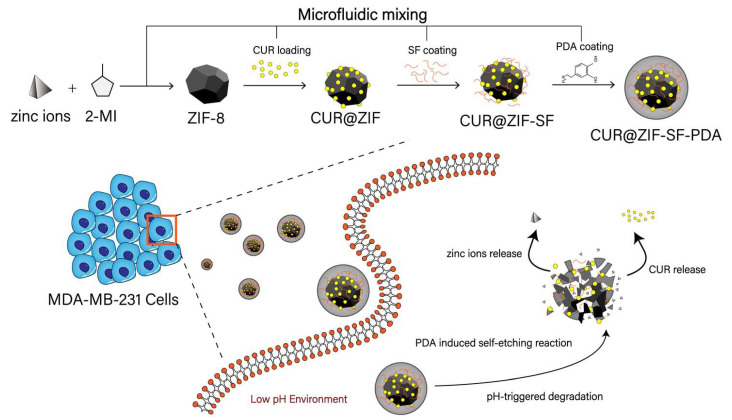
Schematic of ZIF-8-based nanoparticles through the microfluidic method.

ZIF-8-based nanoparticles were prepared, as shown in Figure 2, in which the novel microfluidic design showed a significant control on the properties of synthesized nanoparticles. The formation of ZIF-8 nanoparticles included several steps. Firstly, a ZIF diffusing species is formed through the coordinate reaction between diffused 2-MI and zinc ions, then the nucleation step starts once the local concentration of diffusing species exceeds a certain threshold and this is followed by a growth step to obtain finished ZIF-8 crystals [60]. Therefore, a rapid and homogeneous diffusion of 2-MI, zinc ions, and obtained ZIF species at the beginning is crucial to the kinetics of the nucleation and growth step. For the first time, this novel microfluidic design was used in the preparation of ZIF-8 nanoparticles, with the advantage to provide rapid mixing during ZIF-8 nanoprecipitation. The effectiveness and, for a given mixer size, the speed of the mixing, are dependent on the flow regime. For the swirl mixer, below about Re = 50, inertia is minor, the swirl effect is weak, and mixing in each element is poor. Mixing effectiveness increases with increasing flow rate (and Re)—first, with unsteady flow beginning just above a Re of 100, and, eventually, with fully developed turbulent flow beyond Re around 5000, where the flow undergoes continuous irregular fluctuation of a complex pattern with no distinct streamlines [61,62,63]. The transitional flow regime may perhaps be viewed as progressing from first occurrence of unsteadiness up to fully turbulence, so, it is in the range of Re 150 to 5000. The novel microfluidic device used in this study generated laminar flow and transitional flow by simply increasing the total flow rate from 1 mL/min to 50 mL/min (Re increased from 42 to 2080) (Table 2). Another parameter describing the mixing system—flow mixing time ($\tau_{m}$)—was dramatically reduced from 36.7 ms to 0.7 ms with increasing total flow rate. Therefore, a high total flow rate improved the diffusion pattern of 2-MI, zinc ions, and ZIF species by enhancing irregular chaotic fluctuations during fluid mixing. In addition, a short $\tau_{m}$ enabled ZIF diffusing species to reach a high supersaturation condition rapidly, allowing uniform ZIF-8 nanoparticles to grow simultaneously.

#### 3.1.2. Microfluidic-Controlled Properties of ZIF-8 Nanoparticles

**Figure 3:**
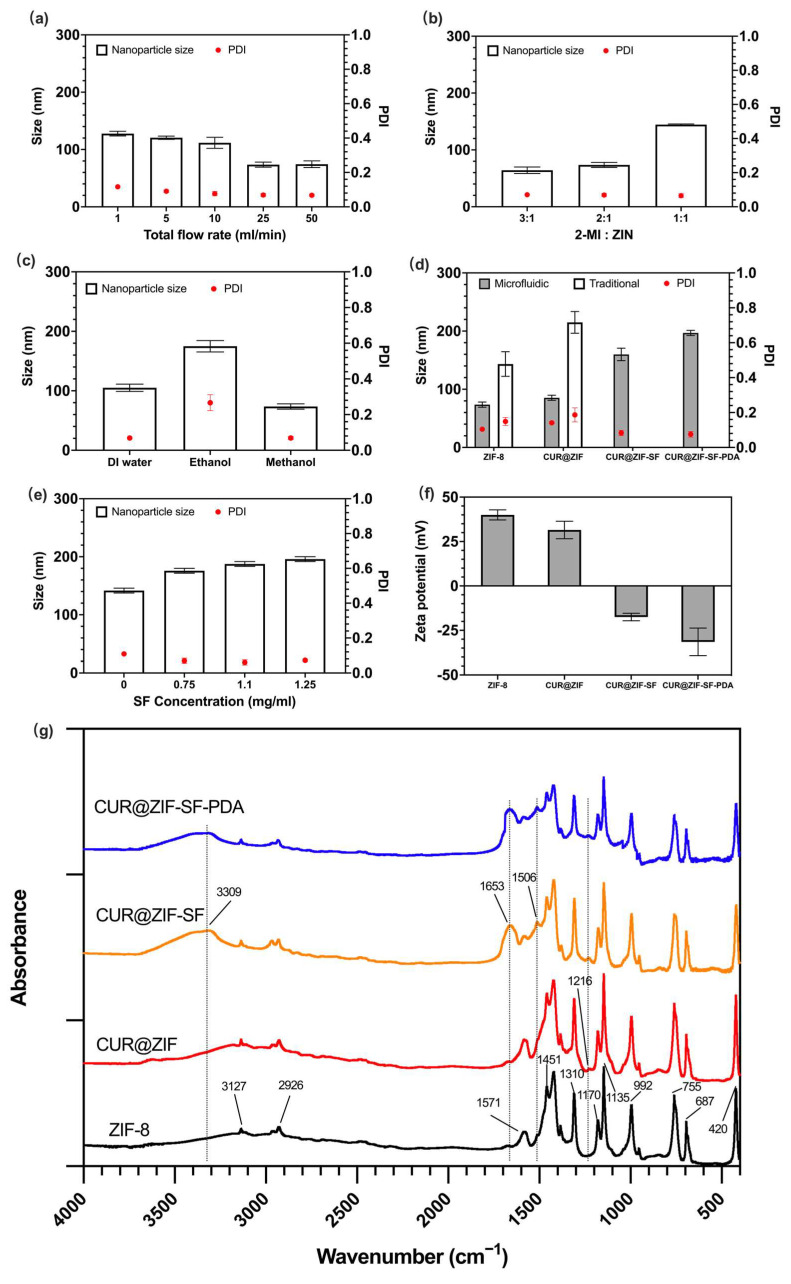
Characterization of designed nanoparticles. The effects of different (**a**) total flow rates (2-MI/ZIN ratio was kept at 2:1), (**b**) 2-MI/ZIN ratios (total flow rate was kept at 25 mL/min), and (**c**) solvents (2-MI/ZIN ratio and total flow rate were kept at 2:1 and 25 mL/min, respectively) on the size and PDI of ZIF-8 nanoparticles. (**d**) The change in size and PDI after each modification step through microfluidic and traditional methods. (**e**) The effect of SF concentrations on the size and PDI of CUR@ZIF-SF-PDA nanoparticles. (**f**) The change in zeta potential after each modification step. (**g**) FTIR spectra of synthesised nanoparticles.

To further evaluate the performance of microfluidic mixing, various total flow rates from 1 mL/min to 50 mL/min were carried out during the preparation of ZIF-8 nanoparticles (methanol was used as 2-MI solvent). The flow rate ratio remained constant: 2-MI solution: ZIN solution = 2:1. Using a higher total flow rate demonstrated a smaller mean particle size and narrower size distribution (lower PDI value) (Figure 3a). The results are consistent with the Reynolds number value observed for each flow rate tested, which showed that increasing the total flow rate and Reynolds number improved the diffusion pattern of mixing, allowing a smaller particle size with narrower size distribution. As indicated in Table 2, the lowest flow rate of 1 mL/min is clearly in the steady laminar flow regime, and the 2-MI and ZIN solutions are unevenly mixed, resulting in a relatively large nanoparticle size. With an increasing total flow rate, the flow entered the unsteady flow regime, in which viscous effects, diffusion layer thickness, and mixing time were decreased, leading to significantly improved mixing efficiency. As seen from Figure 3a, the size of the ZIF-8 nanoparticles decreased from 127 nm to 73 nm with the increased total flow rate from 1 mL/min to 25 mL/min. However, the size was slightly increased from 73 nm to 74.5 nm with the increased total flow rate from 25 mL/min to 50 mL/min. This suggests that, beyond a certain flow rate, the mixing efficiency gradually decreases. It also should be noted that the size distribution of desired nanoparticles could be influenced by the shear stress in microfluidic channels [64,65]. The shear-induced fragmentation or aggregation was found to be influenced by a complicated balance between hydrodynamic and colloidal forces [66]. Shear stress is directly proportional to the total flow rate [67]. In this study, when total flow rate ranged from 1 mL/min to 25 mL/min, the dominant factor affecting the particle size distribution was hydrodynamic force-induced fragmentation. Under these conditions, shear stress led to the breakup of ZIF-8 nanoparticles, resulting in a reduction in particle size. However, when the total flow rate exceeded 25 mL/min, colloidal force induced aggregation became the dominant factor affecting the particle size distribution. In this case, ZIF-8 nanoparticles were more likely to collide and aggregate, resulting in larger particle size. Figure 3b indicates the influence of different flow ratios on the size of ZIF-8 nanoparticles at the fixed total flow rate of 25 mL/min, and methanol was used as the 2-MI solvent. A high 2-MI solution flow rate facilitated the formation of smaller particle size. Based on this result, the excess 2-MI might attach to the surface of synthesised ZIF-8 nanoparticles and prevent further growth and aggregation between particles. Yamamoto et al. [68] showed that a low 2-MI/ZIN mixing ratio resulted in unwanted linking between 2-MI and zinc ions after the nucleation step of ZIF-8 nanoparticles, leading to a large size distribution. On the other hand, when the total flow rate was fixed, the system viscosity was decreased with an increased 2-MI component from 1:1 to 3:1, leading to a higher Re at a larger 2-MI/ZIN ratio (the Re at 2-MI/ZIN ratio = 3:1, 2:1, and 1:1 were 1065, 1040, and 996, respectively), and this could further enhance the rapid mixing effect. Inspired by this result, we analysed the influence of system viscosity on particle size by changing different 2-MI solvents at the fixed total flow rate of 25 mL/min and flow rate ratio of 2-MI solution: ZIN solution = 2:1. Figure 3c shows that the size of ZIF-8 nanoparticles was much smaller when using methanol and DI water as the 2-MI solvent compared with using ethanol, and that was in line with our expectations, as the methanol and DI water used as 2-MI solvent have lower viscosity and larger Re, therefore improving the rapid mixing, while ethanol used as 2-MI solvent, with its higher viscosity, did the opposite [69]. Although DI water used as 2-MI solvent has a lower viscosity and a larger Re compared with methanol, the size of ZIF-8 nanoparticles generated from the DI water mixing system was a little larger than that from the methanol mixing system. The reason for this could be that the DI water used as 2-MI solvent has a greater ability to provide hydrogen bonds (α = 1.17) compared with methanol (α = 0.98), which promoted the deprotonation process of 2-MI, inducing a faster growth of ZIF-8 nanoparticles [70]. Considering CUR loading needs organic solvent, methanol was chosen for further analysis. By strictly controlling the total flow rate (25 mL/min), 2-MI/ZIN ratio (2:1), and the type of solvent (methanol), the designed microfluidic mixing system provides homogenous populations of ZIF-8 nanoparticles for producing stable and efficient drug delivery systems. 

#### 3.1.3. Microfluidic Controlled Properties of CUR@ZIF-SF-PDA Nanoparticles

**Figure 4:**
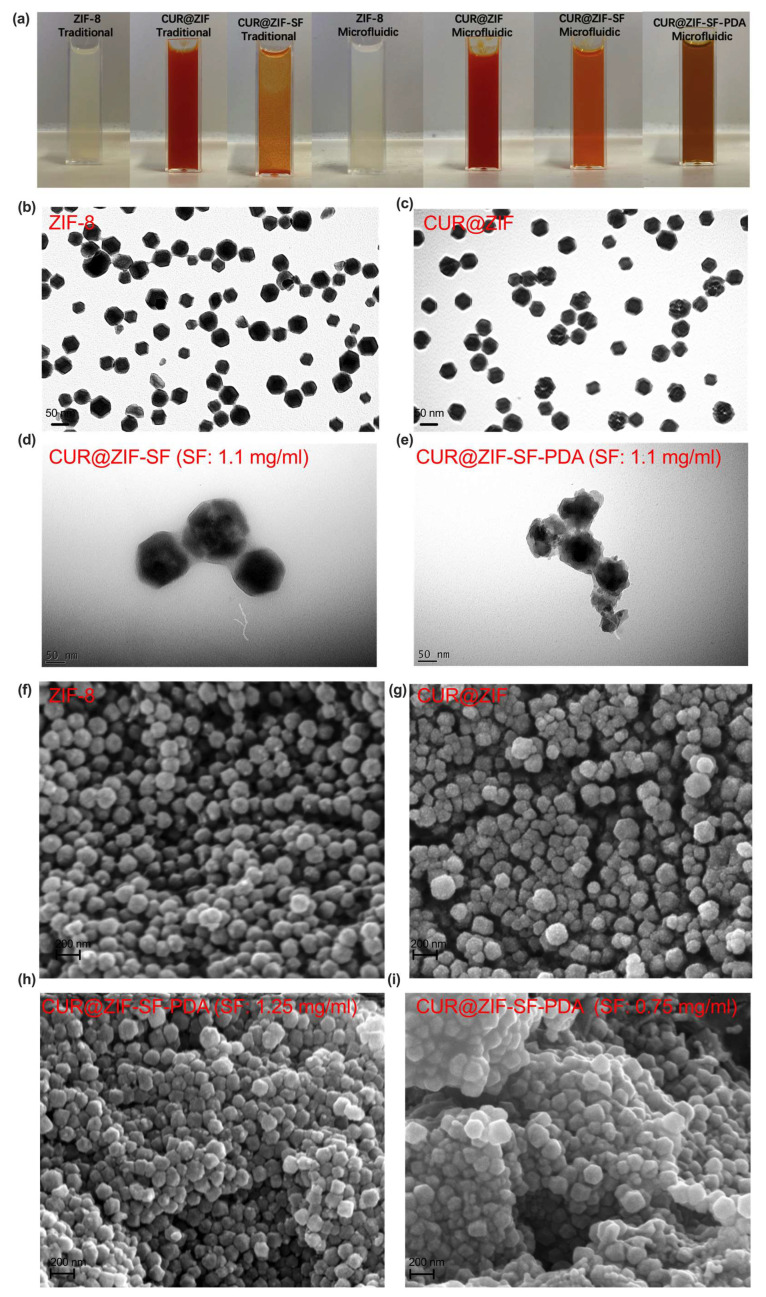
Representative morphological images of the designed nanoparticles. (**a**) Colour change and dispersibility of synthesised nanoparticles through traditional and microfluidic methods. (**b**–**e**) TEM images (scale bar: 50 nm) of microfluidic-assisted nanoparticles taken from FEI Tecnai G2 Spirit BioTWIN with accelerating voltage at 80 kV and (**f**–**i**) SEM images (scale bar: 200 nm) of microfluidic-assisted nanoparticles taken from FEI Inspect F50 Microscope operated at 10 kV.

The novel-designed microfluidic mixing system was not only used for ZIF-8 nanoparticle preparation, but also applied in effective CUR encapsulation and the SF/PDA coating process. Figure 3d illustrates the size change after CUR loading and SF/PDA coating. It can be seen that particle size increased by 12 nm, 75 nm, and 37 nm after CUR encapsulation and SF/PDA coating, respectively, through the microfluidic mixing method. To investigate the differences between the microfluidic and traditional mixing systems, CUR@ZIF-SF-PDA nanoparticles were prepared again in the same way, but using a magnetic stirrer as a mixing method. Here, the size of traditionally prepared particles significantly increased by around two times compared with the microfluidic-assisted method. Interestingly, it was impossible to prepare nanoscale particles of CUR@ZIF-SF/PDA traditionally, as a severe aggregation happened after SF/PDA coating (Figure 4), resulting in much larger size of particles (>1000 nm). As a result, CUR@ZIF-SF/PDA particles prepared by traditional mixing method were not displayed in Figure 3d as nanoparticles. A possible rationale for this aggregation could be the long traditional mixing time (5 s). SF coating relies on the electrostatic adsorption between positively charged ZIF-8 surface and negatively charged SF material, thus the zeta potential of the system gradually changed from positive to negative, in which an unstable electroneutral state is obtained (Figure 3f and Supplementary Figure S1). Rapid mixing provided by a microfluidic mixer significantly shortens this process to milliseconds (ms), therefore preventing the aggregation between neutralized particles. While insufficient mixing during the traditional method allowed an electroneutral state to exist for a long time, the resultant low zeta potential was not strong enough to prevent aggregation from Van der Waals attractive forces, therefore resulting in a large amount of precipitate. To control the release behaviour of zinc ions, various concentrations of SF were prepared as an intermediate protection layer. As shown in Figure 3e, owing to microfluidic rapid mixing, increasing the concentration of SF resulted in a diameter increase from 142 nm to 196 nm without obvious aggregation. Meanwhile, Figure 3f indicates the change in zeta potential from 40 mV to −32 mV after relevant modifications, indicating the successful CUR loading and SF/PDA coating. In addition, their relative large zeta potential (over ±30 mV) could provide a strong electrostatic repulsion between individual particles, leading to a stable nanoplatform [71]. Supplementary Figure S4 illustrates the stability analysis of desired CUR@ZIF-SF-PDA nanoparticles. The results of the size and PDI showed no significant difference after five days of freezing at −20 °C, indicating high stability of desired nanoparticles. Hence, we conclude that microfluidic rapid mixing played an important role in building CUR@ZIF-SF-PDA nanoparticles with suitable charge densities and controllable particle sizes.

#### 3.1.4. Fourier Transform Infrared Spectroscopy (FTIR) Analysis

FTIR spectroscopy was used to investigate the chemical compositions of synthesised nanoparticles (Figure 3g). ZIF-8 nanoparticles showed high-frequency peaks at 3127 cm^−1^ and 2926 cm^−1^ representing the stretching of the aromatic C–H and aliphatic C–H bonds from the imidazole ring, respectively [72]. The peak at 1571 cm^−1^ was assigned to C=N stretch modes, and the bands at 1310–1451 cm^−1^ corresponded to entire ring stretching. Three sharp absorption peaks at 1170 cm^−1^, 1135 cm^−1^, and 992 cm^−1^ were observed for the plane bending of the imidazole ring [73]. Moreover, peaks at 755 cm^−1^ and 687 cm^−1^ belonged to aromatic sp^2^ C–H bending, and a strong peak observed at 420 cm^−1^ was associated with Zn–N stretching mode [74,75]. The peaks mentioned above were presented in all synthesised nanoparticles, indicating that ZIF-8 was used as a core during the modification of nanoparticles. A small new peak at 1216 cm^−1^, corresponding to the in-plane bending of aromatic CCH, was observed after encapsulation of CUR (CUR@ZIF). Compared with CUR@ZIF nanoparticles, SF coating (CUR@ZIF-SF) resulted in three new peaks at 3309 cm^−1^, 1653 cm^−1^, and 1506 cm^−1^, which could be assigned to N–H stretching vibration from amide groups, C=O stretching from SF amide I structure and N–H in-plane bend from SF amide II structure, respectively, indicated the successful coating of the SF intermediate layer [76,77]. In addition, PDA coating led to a significant increase in the absorbance intensity in the range from 1700 cm^−1^ to 1000 cm^−1^, caused by the C–O stretching of the phenol group and stretching vibrations of the aromatic rings. Together, these results imply that the synthesised nanoparticles had been successfully modified. 

### 3.2. Morphological Analysis

Representative images of traditional/microfluidic-assisted nanoparticles were shown in Figure 4a. The colour change from milk-white to red indicated the successful loading of CUR. As mentioned before, traditionally prepared CUR@ZIF-SF nanoparticles had an obvious aggregation, while microfluidic-assisted nanoparticles still maintained a good dispersion, with a colour change from red to orange. Due to the provided alkaline environment (pH = 8.5), the catechol group of dopamine (DA) was easily oxidized and transferred to PDA through a self-polymerization with the colour change from orange to dark brown. The morphology of the designed nanoparticles was inspected by TEM and SEM (Figure 4b–i). Figure 4b,f illustrate that prepared ZIF-8 nanoparticles have a rhombic dodecahedral shape with a diameter of around 65 nm, which is in line with the DLS result (74 nm), as the hydrodynamic diameter detected by DLS includes not only the particle size, but also a liquid layer attached to the particles [78]. Compared with the smooth surface of ZIF-8 nanoparticles, some small dots are observed (Figure 4c,g) after the encapsulation of CUR, indicating that the CUR had been successfully loaded. Figure 4d clearly shows that CUR@ZIF-SF nanoparticles prepared by 1.1 mg/mL of SF still maintain their crystallographic facets because of the protection of SF coating. While, as shown in Figure 4e, a further PDA coating resulted in a collapse of its original crystal shape, indicating a self-etching reaction had been induced between the catechol group from DA and zinc ion nodes. The self-etching procedure will eventually lead to the disintegration of the original ZIF-8 structure because the catechol moiety has a higher binding affinity with zinc ions compared with 2-MI [35]. To further reveal the influence of the SF protection layer on the self-etching effect, different concentrations of SF (0.75 mg/mL and 1.25 mg/mL) were used during CUR@ZIF-SF-PDA nanoparticle preparation. It can be seen from the Figure 4h,i that a higher SF concentration (1.25 mg/mL) could attenuate the self-etching effect by building a thicker SF intermediate layer, preventing the catechol group from grabbing zinc ions from the ZIF-8 framework, while a lower SF concentration (0.75 mg/mL) did not have a significant blocking effect leading to an obvious collapse of the original crystallographic structure. These results suggest that the self-etching reaction could be adjusted by simply changing the concentration of the intermediate SF layer. 

### 3.3. Microfluidic-Controlled Encapsulation/Loading and pH-Responsive Release of CUR and Zinc Ions 

#### 3.3.1. Encapsulation/Loading of CUR and Zinc Ions

**Figure 5:**
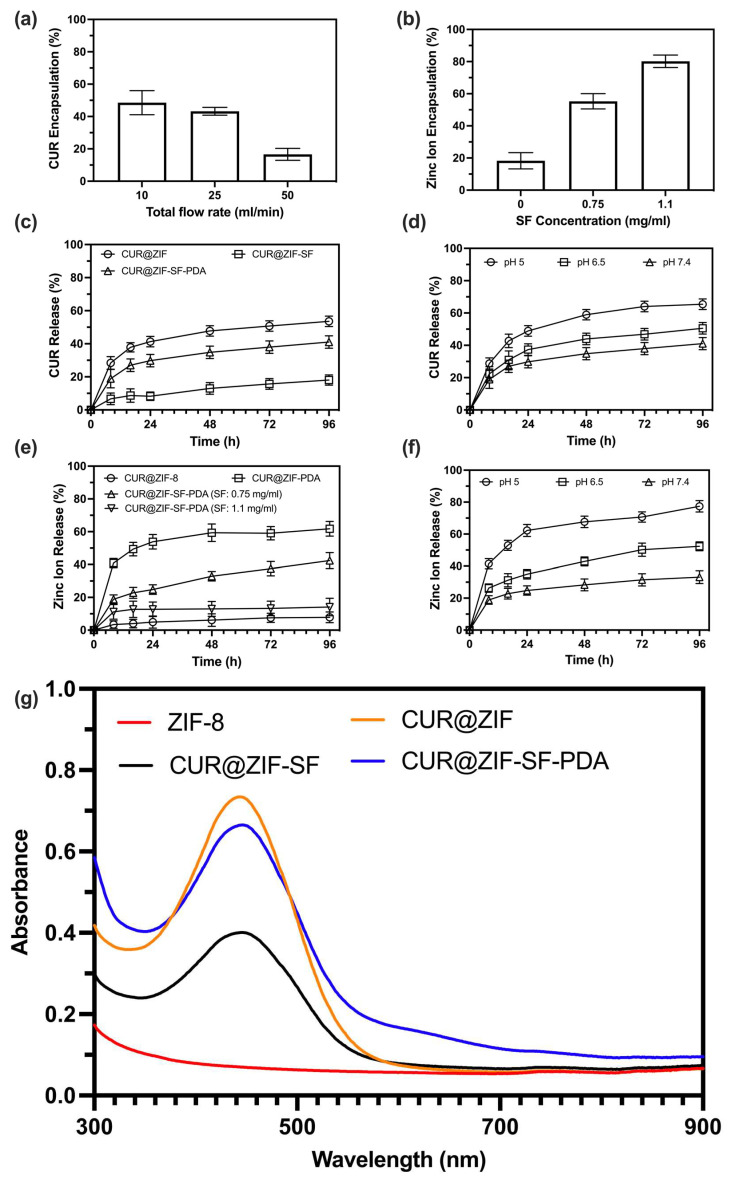
Encapsulation efficiency and release profiles of the designed nanoparticles. (**a**,**b**) The encapsulation efficiency of CUR and zinc ions with various parameters (total flow rates and SF concentrations). In vitro (**c**) CUR and (**e**) zinc ions release profiles of designed nanoparticles with various modifications incubated in the release medium of pH 7.4. The pH-responsive release of (**d**) CUR and (**f**) zinc ions in different pH of 5, 6.5, and 7.4. (**g**) UV-Vis spectra of designed nanoparticles.

The encapsulation of CUR was confirmed by an increase in the hydrodynamic size from ZIF-8 to CUR@ZIF-SF-PDA nanoparticles (Figure 3d), Zeta potential change (Figure 3f), and FTIR analysis (Figure 3g). To further quantify the loading and release behaviour of CUR on the CUR@ZIF-SF-PDA nanoparticles, UV-Vis spectroscopy was performed (Figure 5g and Supplementary Figure S2). Compared with pure ZIF-8 nanoparticles, CUR-loaded nanoparticles had an obvious absorbance peak at 435 nm, belonging to low energy π–π* excitation of CUR, which could be used to detect the concentration of encapsulated CUR [79]. There are no big differences between the traditional method and microfluidic-assisted method on the UV-Vis spectra of ZIF-8 and CUR@ZIF nanoparticles except that, compared with the traditional method, a little bit higher and sharper absorbance peak at 435 nm generated from microfluidic-assisted method was observed, indicating an improved CUR encapsulation efficiency obtained from the microfluidic-assisted method. Figure 5a and Supplementary Figure S3a revealed the relationship between rapid mixing and the encapsulation/loading efficiency of CUR. A higher total flow rate (50 mL/min), representing a faster mixing, decreased the CUR encapsulation efficiency from 49% to 17% and loading efficiency from 11% to 5% compared with a lower total flow rate (10 mL/min). The reason is that the encapsulation/loading of CUR relies on a flash nanoprecipitation where the hydrophobic CUR was dissolved in the water-miscible 2-MI methanol solution and rapidly mixed with antisolvent (ZIN water solution) so that the nanoprecipitation happened and the precipitated CUR could be encapsulated by the synthesised ZIF-8 nanoparticles at the same time [80]. Therefore, an over-high total flow rate (higher than 50 mL/min) resulted in a very short mixing time (less than 0.7 ms), which was not enough for sufficient nanoprecipitation of CUR, leading to a relative low CUR encapsulation efficiency. On the other hand, an over-low total flow rate (lower than 10 mL/min) may cause insufficient mixing and large particle size. Thus, accurate control of a suitable total flow rate at 25 mL/min was crucial to synthesising CUR@ZIF-SF-PDA nanoparticles, as it provided a relatively small particle size and a homogenous size distribution with a small compromise on CUR encapsulation efficiency (44%) and loading efficiency (8.32%).

Unlike CUR, instead of total flow rate, the encapsulation/loading efficiency of zinc ions was more related to SF intermediate protection layer. Hydrolysis-induced degradation and unwanted etching during unprotected PDA coating leads to low storage of zinc ions during the synthesis of CUR@ZIF-SF-PDA nanoparticles [35,37]. Figure 5b and Supplementary Figure S3b illustrate the effect of SF coating on the encapsulation/loading of zinc ions. The absence of the SF protection layer resulted in a large loss of zinc ions (80%) from CUR@ZIF-PDA nanoparticles after PDA coating. This loss could be reduced to 42% and 21% by building SF intermediate layers with various concentrations of SF at 0.75 and 1.1 mg/mL, respectively. Therefore, SF with the ability to tailor the etching reaction between ZIF-8 core and PDA coating could provide physical protection and accurate control of the encapsulation/loading of zinc ions. 

#### 3.3.2. Release of CUR and Zinc Ions

To evaluate the CUR release profile of synthesised nanoparticles, the concentrations of released CUR from CUR@ZIF-8, CUR@ZIF-SF, and CUR@ZIF-SF-PDA nanoparticles were measured at pH 7.4 (Figure 5c). The concentration of SF was fixed at 0.75 mg/mL as its outstanding store and release ability for zinc ions, which will be discussed later. All nanoparticles showed a similar release pattern, in which most of CUR had been released during the first 24 h and followed by a sustained release to 96 h. CUR@ZIF exhibited the highest CUR release at 54%, but, on the contrary, only 18% of CUR was released from CUR@ZIF-SF, which means SF films suppressed the CUR release in the normal physiological environment (pH 7.4). Interestingly, PDA coating resulted in an increase in CUR release from 18% to 42%. This suggested that the collapse of the ZIF-8 framework caused by an etching reaction with PDA films resulted in further release of the CUR encapsulated inside the ZIF-8 core. Figure 5d illustrates the cumulative CUR release curves of CUR@ZIF-SF-PDA nanoparticles at various pH conditions. A higher CUR release (66%) was obtained at pH 5 compared with 51% and 42% of CUR released at pH 6.5 and pH 7.4, respectively. The reason for this could be that the degradation of the ZIF-8 framework is more likely to happen under acidic conditions caused by the protonation of 2-MI, leading to the breakage of coordination between 2-MI and zinc ions [81]. 

Not only CUR, but also the cumulative release of zinc ions, was related to the SF intermediate layer (Figure 5e). Under a physiological environment (pH 7.4), CUR@ZIF nanoparticles showed a minimal release of zinc ions (only 8%) because of no self-etching and acidity-induced release. While after PDA coating, the zinc ions release of CUR@ZIF-PDA was accelerated to 62%, demonstrating the etching reaction between PDA and ZIF-8 was the main driving force of zinc ion release. To further control the release of zinc ions, 0.75 mg/mL and 1.1 mg/mL of SF as an intermediate protection layer was investigated. The result demonstrated that the zinc ions were released in an SF-dependent manner. Specifically, the larger the amount of SF, the more easily it suppressed the release of zinc ions. Interestingly, although CUR@ZIF-SF-PDA nanoparticles with 0.75 mg/mL of SF coating released fewer zinc ions in terms of percentage of loaded zinc ions than CUR@ZIF-PDA nanoparticles, the total amount of released zinc ions from CUR@ZIF-SF-PDA were still larger than that from CUR@ZIF-PDA, as more zinc ions had been previously stored in CUR@ZIF-SF-PDA nanoparticles (under SF layer protection) compared with CUR@ZIF-PDA nanoparticles (without SF layer protection). In addition, instead of releasing most zinc ions within the first 24 h, 0.75 mg/mL of SF coating allowed a more sustained release until 96 h compared with other conditions. As shown in Figure 5f, designed CUR@ZIF-SF-PDA nanoparticles (SF: 0.75 mg/mL) also exhibited a pH-responsive release of zinc ions with a maximum release at 78% in an acidic environment (pH 5). Thus, the designed CUR@ZIF-SF-PDA nanoparticles can be used as promising and efficient pH-responsive nanocarriers for CUR and zinc ions in the acidic tumour microenvironment.

### 3.4. In Vitro Cellular Uptake Analysis

**Figure 6:**
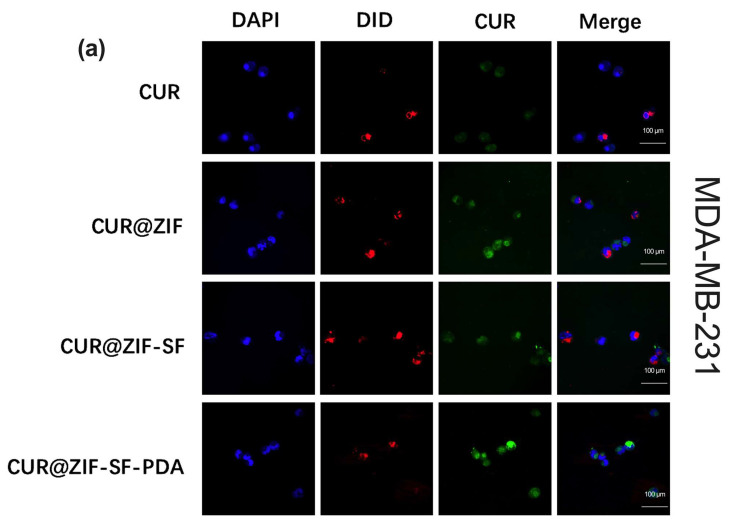

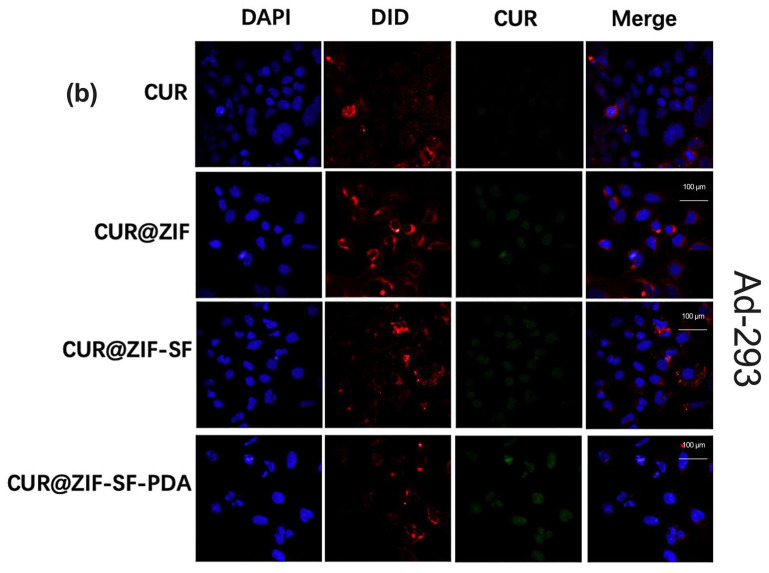
Cellular uptake analysis of designer nanoparticles. Representative fluorescence images of (**a**) MDA-MB-231 human breast cancer cells and (**b**) AD-293 human embryonic kidney cells treated with CUR, CUR@ZIF, CUR@ZIF-SF, and CUR@ZIF-SF-PDA nanoparticles for 24 h. Of note, experiments were repeated three times with the same results. The cell nucleus and cytoskeleton were stained by DAPI (blue) and DiD (red), respectively. The pictures were taken with a Zeiss LSM 980 microscope with an exposure time of 10 s.

To investigate the intracellular uptake and localization of the designed nanoparticles, MDA-MB-231 human breast cancer cells and AD-293 human embryonic kidney cells were treated with free CUR, CUR@ZIF, CUR@ZIF-SF, and CUR@ZIF-SF-PDA nanoparticles for 24 h. The concentration of CUR (50 µg/mL) was set at the same level for each treatment. The cellular uptake of CUR was visualized through confocal microscopy (Figure 6). Green, blue, and red colours represented auto-fluorescent CUR, DAPI-dyed nucleus, and DiD-dyed cytoskeleton, respectively. Due to poor water solubility, hydrophobic CUR suffers from low absorption and poor bioavailability [82]. As presented in Figure 6a, a weak fluorescence of CUR was noticed from free CUR treatment with MDA-MB-231 cells, suggesting poor cellular uptake efficiency. On the contrary, the cells treated with CUR-loaded nanoparticles clearly demonstrated a significant increase in green fluorescence, indicating an enhanced uptake of CUR by MDA-MB-231 cells. Specifically, the fluorescence signal of CUR from CUR@ZIF-SF treatment was slightly weaker than that of CUR@ZIF and CUR@ZIF-SF-PDA treatments, which was consistent with our earlier drug release study that SF coating could partly prevent the release of CUR. A further PDA coating could overcome this by increasing the release ability of the system. Furthermore, the CUR fluorescence signal obtained from free CUR treatment was only observed in the cell cytoskeleton, which was in agreement with the previous study that CUR as a lipophilic drug was more likely to be passively diffused and accumulated in the cell membrane [83]. In contrast, the CUR signals of the designed nanocarriers were distributed in both cytoplasm and nucleus of the cells. This is likely a result of an active transport method (endocytosis), during which the nanoparticles and loaded CUR were transported by enclosing them in vesicles grabbed from the cytoplasmic membrane [84]. To investigate the differential impact of the designed nanoparticles on healthy cells compared to cancer cells, AD-293 human embryonic kidney cells were treated with the same nanoparticles as shown in Figure 6b. The uptake efficiency of CUR in AD-293 cells was found to be significantly lower than that in MDA-MB-231 cells, indicating a reduced cellular uptake efficiency of the desired nanoparticles in healthy cells. Consequently, a sustained and targeted release of CUR was performed, specifically within the breast cancer cells, facilitating the transfer of CUR to the cell nucleus, indicating an improved cellular uptake efficiency in breast cancer cells.

### 3.5. Biocompatibility and In Vitro Cytotoxicity Analysis

**Figure 7:**
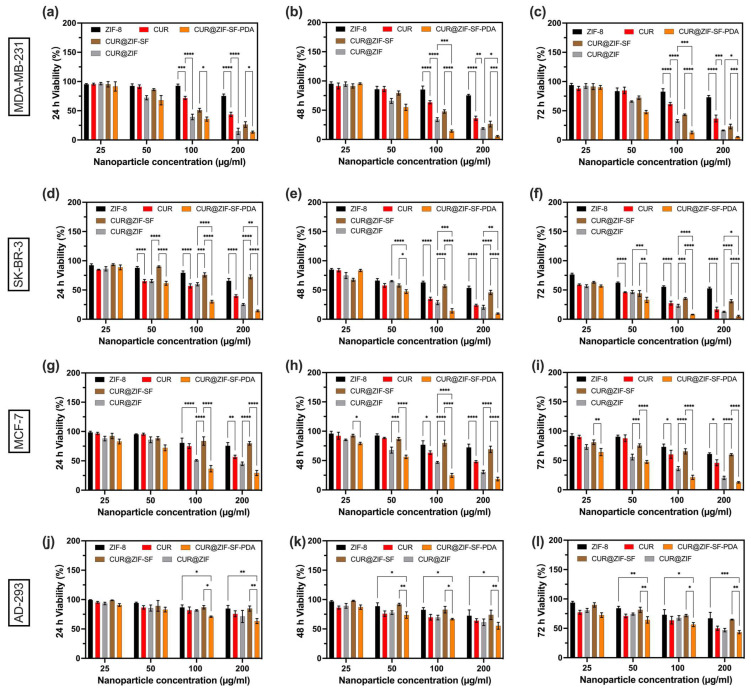
In vitro cytotoxicity analysis of different concentrations of ZIF-8, CUR@ZIF, CUR@ZIF-SF, CUR@ZIF-SF-PDA nanoparticles, and free CUR on (**a**–**c**) MDA-MB-231, (**d**–**f**) SK-BR-3, and (**g**–**i**) MCF-7 human breast cancer cells and (**j**–**l**) AD-293 human embryonic kidney cells after 24, 48, and 72 h of incubation. (* *p* < 0.03, ** *p* < 0.002, *** *p* <0.0002, **** *p* <0.0001).

CUR is a natural compound extracted from curcuma longa, which has proven effective anti-inflammatory and anti-cancer properties [85]. Zinc ion is a crucial mineral nutrient, which plays an important role in the function of enzymes, gene expression, and signal transduction, while an excess of zinc ions leads to cell cytotoxicity by inducing ROS production [86]. ZIF-8 related nanoparticle as the container of zinc ions has proven excellent biocompatibility in various cell lines [75,87,88,89]. In this study, the therapeutic efficiency of the designed system was improved by accurate control of the release properties of zinc ions and CUR through the microfluidic-assisted preparation method. The potential therapeutic efficiency of ZIF-8, CUR@ZIF, CUR@ZIF-SF, CUR@ZIF-SF-PDA nanoparticles, and free CUR was measured by the MTT assay following treatments of MDA-MB-231, SK-BR-3, and MCF-7 breast cancer cells. These cell lines are derived from different breast cancer subtypes (MDA-MB-231: Claudin-low, SK-BR-3: HER2, and MCF-7: Lumical A). AD-293 human embryonic kidney cells (non-cancer cells) were employed to identify the biocompatibility of designed nanoparticles. Figure 7a–l illustrates the change in cell viability after 24, 48, and 72 h of incubation. It can be observed that all treatments exhibited increased cytotoxicity with increased concentration and incubation time. There is only a slight decrease in cell viability after 48 h, as most of the CUR and zinc ions were released within the first two days (Section 3.3.2). During the treatment of MDA-MB-231 cells, for pure ZIF-8 nanoparticles, the cellular viability was maintained over 73% even at a high concentration of 200 µg/mL after 72 h, indicating pure ZIF-8 nanoparticles had great biocompatibility due to their capability to hold zinc ions and presented low cytotoxicity. CUR@ZIF nanoparticles showed significantly higher cytotoxicity compared with free CUR, considering the low cytotoxicity of pure ZIF-8, and this difference could be due to the enhanced delivery of CUR through endocytosis of the designed nanocarriers. Consistent with previous results, SF coating (CUR@ZIF-SF) blocked the delivery of some CUR and zinc ions leading to higher cell viability compared with CUR@ZIF, but it still maintained higher cytotoxicity than free CUR. In the case of CUR@ZIF-SF-PDA nanoparticles, PDA coating did not differ compared with CUR@ZIF in the first 24 h, while after 48 h, the cytotoxicity of CUR@ZIF-SF-PDA nanoparticles (viability was 5.4% at 200 µg/mL) was increased to almost three-fold greater than that of CUR@ZIF nanoparticles (viability was 15.8% at 200 µg/mL). It is expected that PDA coating-induced self-etching reaction resulted in a sustained release of zinc ions inside MDA-MB-231 cells for 72 h, thus allowing a continuous killing of cancer cells. This sustained-release behaviour is crucial for an efficient drug delivery system in which the initial therapeutic dose could be reduced and maintained at the same level [90]. In addition, results indicated the designed nanoparticles induced cytotoxicity on SK-BR-3, and MCF-7 breast cancer cells showed a similar trend compared with MDA-MB-231 breast cancer cells. The cytotoxic effects of CUR, and CUR-loaded nanoparticles on MCF-7 cells, were slightly weaker than those on MDA-MB-231 and SK-BR-3 cells, which were in line with previous research that MDA-MB-231 and SK-BR-3 cells were more sensitive to CUR than MCF-7 cells [91]. A study of the differential susceptibilities of MDA-MB-231 and MCF-7 cells to the CUR’s cytotoxic effects found that the PI3K/Akt-SKP2-Cip/Kips was a crucial pathway modulated by CUR in response to high sensitivity in MDA-MB-231 cells and low sensitivity in MCF-7 cells [92]. Low cytotoxicity on non-cancer cells is an important consideration in drug delivery applications. During the treatment of AD-293 human embryonic kidney cells, most of the cells were alive during the 72 h culture period. The inhibitory concentration (IC_50_) of CUR@ZIF-SF-PDA nanoparticles to kill AD-293 cells remained over 200 µg/mL, even after 72 h of treatment, which was much higher than that of MDA-MB-231, SK-BR-3, and MCF-7 cells at 47 µg/mL, 34 µg/mL, and 49 µg/mL, respectively. Overall, results indicated that the designed nanoparticles had good biocompatibility, with very low cytotoxicity on the non-cancer cells, and showed excellent anti-cancer activity on various breast cancer cell lines.

### 3.6. Cell Cycle Analysis

**Figure 8:**
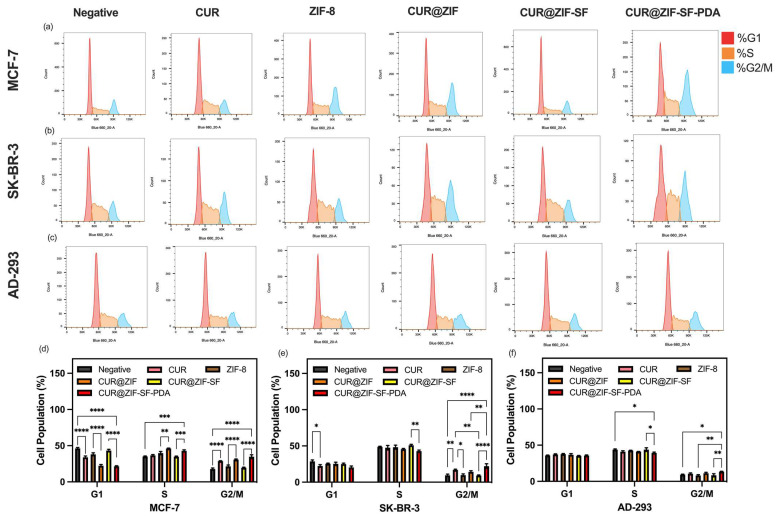
Flow cytometry cell cycle analysis of MCF-7, SK-BR-3 breast cancer cells, and AD-293 human embryonic kidney cells treated with negative control and 100 µg/mL of ZIF-8, CUR@ZIF, CUR@ZIF-SF, CUR@ZIF-SF-PDA, and free CUR for 24 h of incubation. (* *p* < 0.03, ** *p* < 0.002, *** *p* < 0.0002, **** *p* < 0.0001).

The cell cycle plays an important role in the regulation of the cell growth process. DNA damage may cause cell cycle arrest and induce cell death through apoptosis [93]. Figure 8a–c depicts the cell cycle stage of negative control, 100 µg/mL of ZIF-8, CUR@ZIF, CUR@ZIF-SF, CUR@ZIF-SF-PDA, and free CUR treated MCF-7, SK-BR-3 breast cancer cells, and AD-293 human embryonic kidney cells. It can be observed that, for MCF-7 and SK-BR-3 breast cancer cells, CUR and CUR-loaded nanoparticles have recorded increased cell populations in the G2/M phase, accompanied by decreased cell populations in the G1 phase compared with the negative control group. The degree of up-regulation of the G2/M phase was in line with the degree of CUR and zinc ion release described in Section 3.3.2. Among them, desired CUR@ZIF-SF-PDA nanoparticles with the highest release level of CUR and zinc ions remarkably upregulated the G2/M cell cycle phase of MCF-7 and SK-BR-3 cells by around 96% and 134%, respectively, compared with control cells (Figure 8d,e). The results indicated that the inhibitory effect of desired CUR@ZIF-SF-PDA nanoparticles on the proliferation of MCF-7 and SK-BR-3 cells was correlated with DNA damage-induced G2/M phase arrest. This observation is in line with previous reports that CUR and its derivates demonstrated anti-cancer activity by increasing G2/M cell cycle arrest in various breast cancer cell lines, including T47D and MDA-MB-231 cells [94,95,96]. On the other hand, CUR and CUR-loaded nanoparticles treatments showed less effect on the cell cycle distribution of AD-293 human embryonic kidney cells (Figure 8f) compared with MCF-7 and SK-BR-3 breast cancer cells (Figure 8d,e), suggesting that desired nanoparticles induced less DNA damage on non-cancer cells than that on breast cancer cells in line with the MTT data above.

## 4. Conclusions

In summary, our new core-shell CUR@ZIF-SF-PDA nanoparticles with controlled release property of CUR and zinc ions have been developed successfully. For the first time, microfluidic rapid mixing was used to precisely control the proportion of each component of ZIF-8, CUR, SF, and PDA in a hybrid nanoplatform to improve the anti-cancer performance. The desired nanoparticles showed a high degree of modifiability on particle size, zeta potential, and drug release behaviour by simply regulating the microfluidic total flow rate and flow rate ratio. SF as an intermediate layer successfully kept ZIF-8 intact during PDA coating and controlled the release of zinc ions induced by the self-etching reaction between ZIF-8 and PDA. The pH-dependent release behaviour further improved the targeted delivery to cancer cells with an acidic microenvironment. In vitro cellular uptake and cytotoxicity results confirmed the cellular internalization and anticancer properties of our designer CUR@ZIF-SF-PDA nanoparticles in various breast cancer cells, owing to their sustained release profile and G2/M phase cell cycle arrest. High biocompatibility allowed low cytotoxicity on non-cancer cells. In the meantime, our data support other studies, which have used nanoparticles for targeting MDA-MB-231 breast cancer cells, including polyethyleneimine (PEI)-modified polylactide (PLA) nanoparticles and poly(cyclohexene phthalate)(CHO/PA) nanoparticles, and these provide a strong rationale for using nanomedicines to treat aggressive breast cancers, such as triple-negative breast cancers (TNBC) [97,98]. Future studies will assess the in vivo efficacy of designed CUR@ZIF-SF-PDA nanoparticles. While this study specifically investigates the anticancer properties of zinc ions and curcumin, it should be noted that these could be substituted with other drugs for cancer treatment. Therefore, this microfluidic-assisted self-etching drug delivery system provides a great opportunity for producing designer nanomedicines with potential anti-cancer properties.

## Figures and Tables

**Table 1:** Various nano-formulations of curcumin in breast cancer treatment.

Nanoparticles	Size (nm)	Zeta Potential (mV)	Cell Line	Loading Efficiency (LE) Encapsulation Efficiency (EE)	Results	Refs.
CUR-loaded chitosan/protamine nanocarrier	85–340	26.66	MCF-7	LE: 40.2% EE: 67%	Significantly enhanced the antitumor efficacy by inhibiting NF-kB, IL-6, TNF-α, and the downregulation of Bcl-2.	[52]
Peptide-HAS/CUR nanoparticle	246.5	−24.5	MDA-MB-231 SK-BR-3 MCF-7	LE: 5.52% EE: 77.8%	Had great potential for the treatment of PDL1-expressing breast cancer cells	[53]
CUR-loaded hyaluronic acid modified mesoporous silica nanoparticle	161.3	−35	MCF-7 MDA-MB-231	LE: 14.76% EE: 18.5%	The nanohybrid exhibited a significant reduction in tumour volume in tumour-bearing mice compared to free curcumin	[54]
CUR diethyl γ-aminobutyrate-loaded chitosan-coated magnetic nanocarriers	135–175	14	MDA-MB-231	LE: 1.6% EE: 96.1%	Enhanced activity when compared to free CUR diethyl γ-aminobutyrate	[55]
CUR- and paclitaxel-loaded PCEC nanoparticles	27.97	−9.4	MCF-7	LE: 6–11.5% EE: 86.3–90.3%	Enhanced inhibition of tumour growth with reduced side effects compared with free CUR and paclitaxel	[56]

**Table 2:** Reynolds numbers (Re) and mixing times ($\tau_{m}$) at different total flow rates.

Total Flow Rate (mL/min)	Re ^1^	$\tau_{m}$ (ms)
1	42	36.7
5	208	7.3
10	416	3.7
25	1040	1.5
50	2080	0.7

^1^ Re was calculated by using methanol as the 2-MI solvent, 2-MI solution: ZIN solution = 2:1.

## Data Availability

Not applicable.

## Supplementary Materials

The following supporting information can be downloaded at: https://www.mdpi.com/article/10.3390/pharmaceutics15071811/s1, Figure S1: Zeta potential of ZIF-8, CUR, SF, and PDA measured by Dynamic Light Scattering (DLS); Figure S2: UV-Vis spectra of ZIF-8 and CUR@ZIF nanoparticles prepared by traditional magnetic stirrer mixing method. CUR@ZIF-SF/PDA particles prepared by traditional magnetic stirrer mixing method were not displayed here as a severe aggregation happened after SF/PDA coating, resulting in millimeter-sized particles; Figure S3: The loading efficiency of (a) CUR and (b) zinc ions with various parameters (total flow rates and SF concentrations); Figure S4: The stability of CUR@ZIF-SF-PDA nanoparticles for 5 days of storage at −20 °C.
