# Supplementary material for: Microfluidic-Assisted ZIF-Silk-Polydopamine Nanoparticles as Promising Drug Carriers for Breast Cancer Therapy
Source: Pharmaceutics. 2023 Jun 24;15(7):1811. doi: 10.3390/pharmaceutics15071811 (PMC10384305; doi:10.3390/pharmaceutics15071811)
Supplement: Supplementary file 1 [file pharmaceutics-15-01811-s001.zip › pharmaceutics-2370244-supplementary.pdf]

Article

Microfluidic-assisted ZIF-Silk-Polydopamine Nanoparticles as Promising Drug Carriers for Breast Cancer Therapy

Zijian Gao, Muhamad Hawari Mansor, Natalie Winder, Secil Demiral, Jordan Maclnnes, Xiubo Zhao, and Munitta Muthana

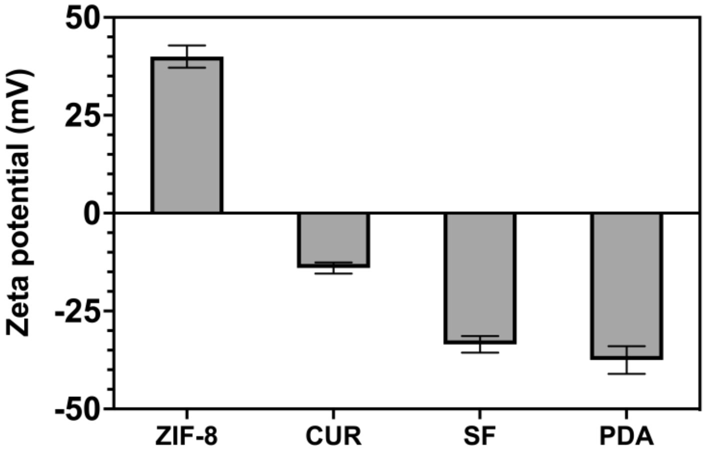

Figure S1. Zeta potential of ZIF-8, CUR, SF, and PDA measured by Dynamic Light Scattering (DLS).

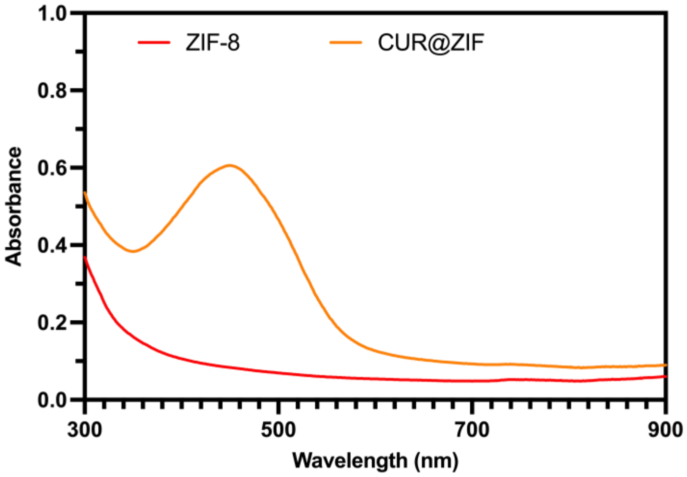

Figure S2. UV-Vis spectra of ZIF-8 and CUR@ZIF nanoparticles prepared by traditional magnetic stirrer mixing method. CUR@ZIF-SF/PDA particles prepared by traditional magnetic stirrer mixing method were not displayed here as a severe aggregation happened after SF/PDA coating, resulting in millimeter-sized particles.

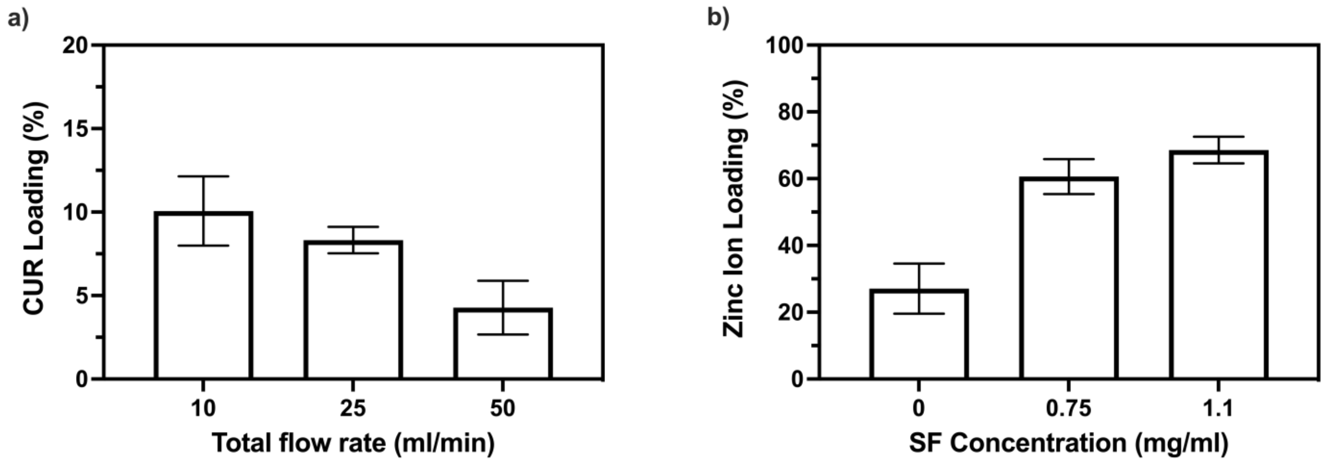

Figure S3. The loading efficiency of a) CUR and b) zinc ions with various parameters (total flow rates and SF concentrations).

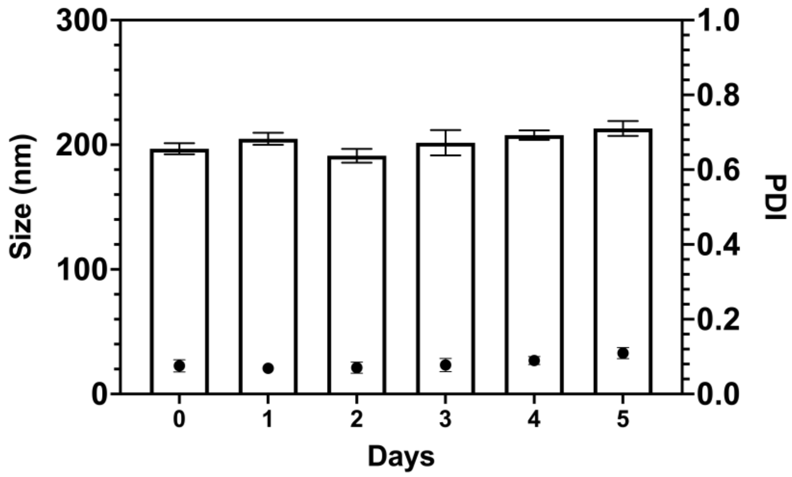

Figure S4. The stability of CUR@ZIF-SF-PDA nanoparticles for 5 days of storage at -20 ℃.
